# Identification of new genetic resources for drought tolerance-related traits from the world *Erianthus* germplasm collection

**DOI:** 10.3389/fpls.2025.1684712

**Published:** 2025-11-28

**Authors:** Valarmathi Ramanathan, Anjan Kumar Pradhan, Prasad Gandham, Appunu Chinnaswamy, H. K. Mahadeva Swamy, K. Mohanraj, Rasitha R, Niranjan Baisakh

**Affiliations:** 1 Division of Crop Improvement, Indian Council of Agricultural Research (ICAR)-Sugarcane Breeding Institute, Coimbatore, India; 2 School of Plant, Environmental and Soil Sciences, Louisiana State University Agricultural Center, Baton Rouge, LA, United States

**Keywords:** drought, *Erianthus*, heritability, marker–trait association, SNP effect

## Abstract

Drought is one of the most complex abiotic stress factors and significantly affects sugarcane production worldwide. With an objective to identify drought-tolerant wild sugarcane germplasm for sugarcane improvement, a set of 223 diverse *Erianthus* clones collected from seven different countries were assessed for drought tolerance-related traits at the formative stage. A drought association panel of 91 *Erianthus* clones with differential drought response was developed for genomic studies to identify marker–trait associations. Drought-tolerant *Erianthus* clones maintained high chlorophyll fluorescence (*F*_v_/*F*_m_) and relative water content and chlorophyll content, and were able to regulate photosynthetic rate as well as canopy cooling to sustain growth and biomass accumulation. They also recorded high stress tolerance indices and a low susceptibility index. Moderate to high heritability estimates of the traits suggested that the traits were under genetic control. A genome-wide association study conducted using 1,044 genotyping by sequencing-derived high-quality single-nucleotide polymorphism markers on the drought panel identified 43 quantitative trait nucleotides (QTNs) associated with various physiological traits that explained phenotypic variance ranging from 0.26% to 59.35%. Candidate genes [Target of Rapamycin 2 (TOR2), Trans-Membrane Kinase 1 (TMK1), potassium transporter auxin response factor, FAD-binding PCMH-type domain-containing protein, and Nitrate Transporter 1/Peptide Transporter Family (NRT1/PTR)] involved in drought perception and signaling were successfully identified in QTN regions. The present study identified drought-tolerant *Erianthus* clones and the QTNs associated with drought tolerance traits for further validation and subsequent utilization in the sugarcane breeding program.

## Introduction

The diverse wild germplasm collection is an important source of desirable alleles contributing to improved yield and resistance to various biotic and abiotic stresses (Fukuhara et al., 2013). Sugarcane (*Saccharum* spp. hybrids) belongs to the genus *Saccharum*, which, along with potential interbreeding genera (*Erianthus, Miscanthus, Narenga*, and *Sclerostachya*), forms the “*Saccharum* complex” and serves as the major wild germplasm for sugarcane (Mukherjee, 1957).

*Erianthus* is the most closely related genus to the *Saccharum* primary gene pool (Mukherjee, 1958; Nair and Mary, 2006). Among seven closely related species of *Erianthus* (*E. arundinaceus*, *E. bengalense*, *E. elephantinus*, *E. hosti*, *E. ravennae*, *E. procerus*, and *E. kanashiroi*), *E arundinaceus* is the most widely distributed species reported from India, Burma, China, Indonesia, Malaysia, New Guinea, Philippines, and Thailand (Mukherjee, 1957; Dao et al., 2013; Tsuruta et al., 2022). All *Erianthus* species are known for their exceptional adaptability to abiotic and biotic stress, high biomass, high tillering ability, and better ratooning ability, which have been attributed to its extensive root system (Nair et al., 2017; Wang et al., 2019; Valarmathi et al., 2020, 2023; Meena et al., 2024; Takaragawa and Wakayama, 2024; Wang et al., 2025).

Intergeneric hybridization of sugarcane with other related genera within the *Saccharum* complex was attempted to broaden the genetic base of modern sugarcane cultivars and introgress more productive and better stress adaptation traits (Nair et al., 2017; Piperidis et al., 2000; Terajima et al., 2022, 2023). Early hybridization attempts between *Saccharum* spp. and *Erianthus* as a potential breeding material for improving sugarcane resulted in limited success due to cross-incompatibility, inherent difficulty in identifying true hybrids from self-progeny, chromosome elimination, and unreduced gamete formation (D’Hont et al., 1995; Piperidis and D’hont, 2001; Piperidis et al., 2010; Jackson and Henry, 2011). However, consequent hybridization efforts led to a few successful intergeneric hybrids between sugarcane and *Erianthus* (Deng et al., 2002; Cai et al., 2005; Piperidis et al., 2010; Chang et al., 2012; Fukuhara et al., 2013; Nair et al., 2017; Pachakkil et al., 2019; Meena et al., 2024). Hybrids developed from the cross between *Erianthus procerus* “IND 90-776” and *Saccharum officinarum* “PIO 96-435” showed higher resistance to red rot with better tolerance to drought (Nair et al., 2017). Progeny of sugarcane and *E. arundinaceus* possessed improved root characteristics and enhanced drought tolerance traits (Fukuhara et al., 2013), resistance to low temperature, and red rot resistance (Ram et al., 2001). *Erianthus*, as a high biomass plant, is also utilized in developing high-fiber energy canes as a bioenergy crop (Deng et al., 2002; Chang et al., 2012; Meena et al., 2024). Several hybridization attempts using *Saccharum* and *E. arundinaceus* as a male parent successfully produced hybrids with desirable traits transferred from *Erianthus* (Sreenivasan et al., 1987; Ram et al., 2001).

Drought is a major abiotic stress factor limiting sugarcane production worldwide (Wang et al., 2003; Dhansu et al., 2021). Most commercial sugarcane varieties, with their shallow root system, are susceptible to moisture stress as well as other belowground extreme conditions (Pierre et al., 2019), which results in reduction in shoot growth, cane yield, and sucrose content (Hoang et al., 2019). World germplasm collections of *E. arundinaceus* clones collected from all over India and other countries such as Burma, Fiji, Indonesia, New Guinea, Pakistan, and Philippines maintained at the Indian Council of Agricultural Research–Sugarcane Breeding Institute, Coimbatore represent a diversified *Saccharum* germplasm resource (Cai et al., 2012; Scortecci et al., 2012; Valarmathi et al., 2021). *Erianthus* species clones with a deeper and prolific root system architecture have been shown to impart drought tolerance by leveraging more underground resources (Narayanan et al., 2014; Valarmathi et al., 2020, 2023). However, sequence variants controlling drought tolerance among *Erianthus* genetic resources have not been identified for their use in targeted sugarcane breeding programs.

Despite the high biomass, good Brix value, ratooning ability, and wide environmental adaptability, genetic studies in *Erianthus* are very limited (Cai et al., 2005; Tsuruta et al., 2012, 2022). On the other hand, genotyping by sequencing (GBS)-derived single-nucleotide polymorphisms (SNPs) have been used for the identification of markers linked to genes associated with various agronomic traits of interest in sugarcane via QTL mapping using biparental or self-population and/or genome-wide association mapping using a diversity panel (Balsalobre et al., 2017; Yang et al., 2018, 2019, 2020; Gutierrez et al., 2018; Fickett et al., 2019; Wirojsirasak et al., 2023; Cortes et al., 2024). The recently available reference genome of the wild progenitor *S.* sp*ontaneum*, sugarcane hybrids, and sequence variations can be better identified at high resolution in sugarcane (Garsmeur et al., 2018; Zhang et al., 2018; Bao et al., 2024; Healey et al., 2024). Some *Erianthus* species such as *E. fulvus* and *E. rufipilus* possess the lowest chromosome number (2n=2x=20) within the *Saccharum* complex (Amalraj and Balasundaram, 2006) and their genome is now available to facilitate genetic studies (Kui et al., 2023; Wang et al., 2023).

In this first ever genome-wide association study, 223 accessions from the world germplasm collection of *Erianthus* maintained at the Sugarcane Breeding Institute, Coimbatore, India were phenotyped for drought tolerance-related traits from 2017 to 2020. Based on the 3 years of field phenotyping data, a drought panel of 91 clones was constructed for a genome-wide association analysis, which identified 43 significant quantitative trait nucleotides (QTNs) and linked genes associated with 12 traits (control and drought stress).

## Materials and methods

### Plant material

A total of 223 germplasm accessions belonging to five different species of the genus *Erianthus* [*E. arundinaceus* (208) clones, *E. bengalense* (3), *E. procerus* (5), *E. ravennae* (3), and *E. elegans* (4)] maintained at the ICAR, Sugarcane Breeding Institute, Coimbatore, India were used in the study. The germplasm included collections from different geographical regions from countries such as Burma, Fiji, India, Indonesia, New Guinea, Pakistan, and Philippines (Valarmathi et al., 2021).

### Experimental site and climatic conditions

The experiment was conducted at the farm field of ICAR Sugarcane Breeding Institute, Coimbatore, India (77°E longitude and 11°N latitude; elevation, 427 m) (1,500–1,800 µmol m^−2^ s^−1^ light intensity, photoperiod of 16 h light and 8 h dark, temperature of 30°C ± 2°C with ~75% relative humidity). The soil at the experimental site was sandy clay loam, taxonomically classified as typic haplustalf with a pH of 7.7. The field site experiences a typical tropical wet and dry climate, experiencing dry season from March to June, intermittent drying from July to September, and wet season lasting from October to December due to the northeast monsoon. Planting was done in December, and the standard agronomic cultural method was followed, which included applications of N, P, and K at 280, 62.5, and 120 kg ha^−1^, respectively. Phosphorus (62.5 kg ha^−1^) was applied as basal dressing before planting, while nitrogen and potassium were applied at 45 and 90 days after planting in two equal splits.

#### Experimental design and drought stress

The field evaluation of drought tolerance of the 223 *Erianthus* accessions was laid out in an augmented block design (ABD) trial for 2 years (2017–2018) along with drought-tolerant (SES 288 and SES 293) and drought-susceptible (IS 76–215 and IS 76 218) checks. From the ABD trial, 91 *Erianthus* clones were selected and evaluated in a randomized block design (RBD) trial for 3 years (2019–2021) in three replications along with the drought-tolerant and -susceptible checks. For both ABD and RBD trials, the clones were planted in 6-m-long rows with 3 m spacing between the rows and 0.5 m spacing between clumps. Two-budded setts of each clone were planted at 12 setts for every 6-m-long row with 3 m spacing between the rows and 0.5 m spacing between clumps. Each plot had 20 rows, 60 m long with 2 m spacing between adjacent plots and 5 m spacing between control and drought blocks.

Drought stress was imposed by withholding irrigation at the formative phase of the crop (60 days after planting). The progress of drought stress was monitored by measuring the soil moisture. The drought trial was left completely under rainfed conditions throughout the crop cycle, and the biomass data were recorded at harvest. The soil moisture content of the experimental field was measured using a pressure plate apparatus (Chakraborty et al., 2010). The soil moisture content of the experimental plot at field capacity was measured in the range of 16.18%–18.45%, and the permanent wilting point was in the range of 7.24%–8.56%.

For the ABD trial, the total rainfall during the formative phase of the crop season for the first year (2017) was 86.5 mm and that for the second year was 288.5 mm. Rainfall was unevenly distributed throughout the cropping season in both years, and the crop faced severe moisture stress with a gradual decline in soil moisture content from below 70% to 40% field capacity during the formative growth stages. For the RBD trial, the total rainfall during the formative phase was 101.4 mm in 2019, 170.5 mm in 2020, and 127.3 mm in 2021. Rainfall distribution was uneven in all three years with severe moisture stress and gradual decline in soil moisture content from 60% to 30% field capacity during the crop’s formative growth stages. There were intermittent drought cycles with below 50% field capacity during grand growth stages. The control field was given regular irrigation at 100% field capacity until harvest. During the stress period, data on morphological and physiological traits were recorded. At harvest, plant height, tiller numbers, and total biomass per clump were recorded under both control and drought conditions.

### Phenotyping during drought stress and field data analysis

During drought, morphological symptoms of leaf drying were recorded through visual scoring. The clones were given a drought score based on the percentage of leaf drying (0%–60%). Physiological data on the leaf relative water content (RWC), canopy temperature, and chlorophyll fluorescence (*F*_v_/*F*_m_) were recorded for the drought-stressed clones and compared with that of control plants. For measuring RWC, approximately 5 cm^2^ of fully expanded leaves were cut and placed in pre-weighted tubes and fresh weight (FW) was recorded. The leaves were saturated in distilled water for 5 h at 4°C in darkness and turgid weight (TW) was recorded. Then, the leaves were air-dried in an oven at 65°C for 48 h and dry weight (DW) was recorded. RWC was calculated using the equation of Schonfeld et al. (1988). Canopy temperature was measured using a handheld infrared gun (Model APOGE MI200) between 11:00 a.m. and 1:00 p.m. Chlorophyll fluorescence (*F*_v_/*F*_m_) was measured during clear sunlight days using the chlorophyll fluorometer OS1P (OPTI-SCIENCES, Hudson, USA). The middle portion of the fully expanded second leaf of the main shoot was dark-adapted using the leaf clip and fluorescence was measured. The variable fluorescence (*F*_v_) represents the difference between *F*_o_ (the minimum fluorescence) and *F*_m_ (the maximum fluorescence). Total chlorophyll content was measured gravimetrically using the method of Arnon (1994). Leaf area (LA) was measured using a non-destructive linear measurement method as mentioned by Montgomery (1911) using the formula LA = *LBK* (cm^2^), where *L* = maximum length of length, *B* = maximum breadth, and *K* = constant (0.75 calculated from the regression analysis). Yield-related traits such as plant height, tiller number, and biomass per clump were recorded at harvest (12 months after planting) for drought-stressed as well as irrigated control plants.

### Statistical analysis

The statistical significance of the morphological traits across seasons was evaluated by estimating variance components using a linear mixed model. For the ABD trial, the lme (linear mixed effect model) package in R v4.4.3 was used to calculate the best linear unbiased predictors (BLUPs), and the BLUP values were used for calculating percent reduction under drought and heritability. For the RBD trial, the restricted maximum likelihood method, implemented in Meta R software, was employed with year as the fixed effect and germplasm and replication as random factors. BLUP-based mean values were subjected to principal component analysis (PCA) using FactoMineR and factoextra packages in R v4.4.3. Broad-sense heritability (%) was estimated in Meta R using the following equation:


$$H^{2}=\;\frac{Vg}{Vg+\;(Verr/r)}$$

where *V*_g_ is the genotypic variance, *V*_err_ is the error variance, and *r* is the number of replications.

### Stress tolerance and susceptibility index

Stress tolerance index (STI), drought tolerance index (DTI), yield index (YI), and stress susceptibility index (SSI) were computed for each germplasm using the following equations.


STI=(Ys×Yp)(Y¯p2)

(Fernandez, 1992)


$$DTI=\left(Y_{s}\times (Y_{s}/Y_{p})\right)/{\bar{Y}}_{s}$$

(Lan, 1998)


$$SSI=\left(1-Y_{s}/Y_{p})\right)/\left(1-({\bar{Y}}_{s})/({\bar{Y}}_{p}))\right)$$

(Fischer and Maurer, 1978)


$$YI=\left(Y_{s}\right)/\left({\bar{Y}}_{s}\right)$$

(Guttieri et al., 2001)

where *Y*_s_ is the mean value of germplasm under stress conditions and *Y*_p_ is the mean value of germplasm under normal conditions.

### Selective genotyping by sequencing of the association panel

A selective panel of 96 *Erianthus* germplasm was constructed based on the drought phenotypic data. The panel comprised drought-sensitive and -tolerant clones and clones with intermediate drought reactions. Approximately 1.5 μg of total genomic DNA with an OD_260_/OD_280_ ratio of 1.8 to 2.0 (assessed using a Qubit 3.0 Fluorometer and NanoDrop 2000 spectrophotometer) from each clone was used for library construction for GBS as described earlier (Gutierrez et al., 2018). Briefly, 0.3–0.6 μg of genomic DNA was digested with *PstI*, ligated with P1 and P2 barcoded adapters followed by PCR amplification. Amplified DNA libraries were quantified using a Qubit^®^ 4.0 Fluorometer, and the insert size was assessed using the Agilent^®^ 2100 Bioanalyzer followed by quantitative real-time polymerase chain reaction (qPCR). Libraries with an appropriate insert size with a concentration of more than 2 nM were pooled and sequenced paired end on an Illumina NovaSeq 6000 platform, with a read length of 144 bp from each end.

### Identification of SNPs

Raw sequence reads were filtered using the FastQC method as described earlier and high-quality reads (score >30) were mapped against the *Erianthus rufipilus* (https://sugarcane-genome.cirad.fr/node/1/7) genome (Wang et al., 2023) using bcftools, FreeBayes, and GATK version v4.1 to identify nucleotide variants (SNPs) as described earlier (Shahi et al., 2025). SNPs commonly identified by the three software were filtered at >0.1 minor allele frequency and <50% missing data.

### Population structure

To estimate the number of populations, the association panel was assessed using discriminant analysis of principal components (DAPC) with the “adegenet” package of R v4.4.3 (Jombart et al., 2010). Structure analysis was performed with SNPs having high polymorphic information content using the admixture model of STRUCTURE v2.3.4 (Pritchard et al., 2000). The *K* value (i.e., no. of sub-populations) was set at 2–10 with Monte Carlo Markov chain (MCMC) simulation and a burn-in of 20,000 iterations. The corresponding population membership was used as the Q-matrix for marker–trait association analysis. Furthermore, the genetic structure of the lines was determined by cluster analysis based on the shared-allele distance using the neighbor-joining tree algorithm in TASSEL v.5.29 where the branching pattern was assessed with bootstrapping.

### Linkage disequilibrium

LD between each pair of markers was estimated as squared allele frequency correlation (*r*^2^) using TASSEL v.5.29 (Bradbury et al., 2007). LD decay distance was estimated by plotting the *r*^2^ values between marker pairs against physical distance. The intersection points of the LOESS curve and the *r*^2^ threshold in the scatter plot was identified as the average LD decay across the genome.

### Genome-wide association analysis

A genome-wide association study (GWAS) was conducted using the best linear unbiased estimates (BLUEs) of the phenotypic data collected across 3 years (2021–2023) of a panel comprising 96 *Erianthus* clones and 1,044 high-quality SNPs to identify QTNs associated with morpho-physiological traits under drought stress. GWA analysis was conducted in the R package GAPIT v.3 (Wang and Zhang, 2021) using the multi-locus models such as fixed and random model circulating probability unification (FarmCPU), Bayesian-information and linkage-disequilibrium iteratively nested keyway (BLINK), and multiple loci mixed model (MLMM) with default parameters. False discovery rate (FDR) < 5% (Benjamini and Hochberg, 1995) was used as the threshold to determine significant QTNs. Furthermore, six other multi-locus GWAS models, such as mrMLM, FASTmrMLM, FASTmrEMMA, pLARmEB, ISIS EM-BLASSO, and pkWmEB, were implemented in the R package mrMLM v5.0 (https://cran.r-project.org/web/packages/mrMLM/index.html) with default parameters. The QTNs with LOD score ≥3.00 were declared significant. A QTN identified in at least two models was designated as a consistent QTN, and a QTN observed in three or more models with >10% phenotypic variation explained (PVE or *R*^2^) at a *p*-value < 0.00001 was considered as a major QTN.

### Identification of candidate genes

Genes within 200-kb flanking regions of the significant QTNs were retrieved from the *E. rufipilus* reference genome (https://sugarcane-genome.cirad.fr/node/1/7). Downstream gene ontology (GO) enrichment analysis was performed using two gene annotation databases DAVID (https://davidbioinformatics.nih.gov) and agriGO V2.0 (http://systemsbiology.cau.edu.cn/agriGOv2/).

## Results

### Comparative drought performance of *Erianthus* clones

**Figure 1:**
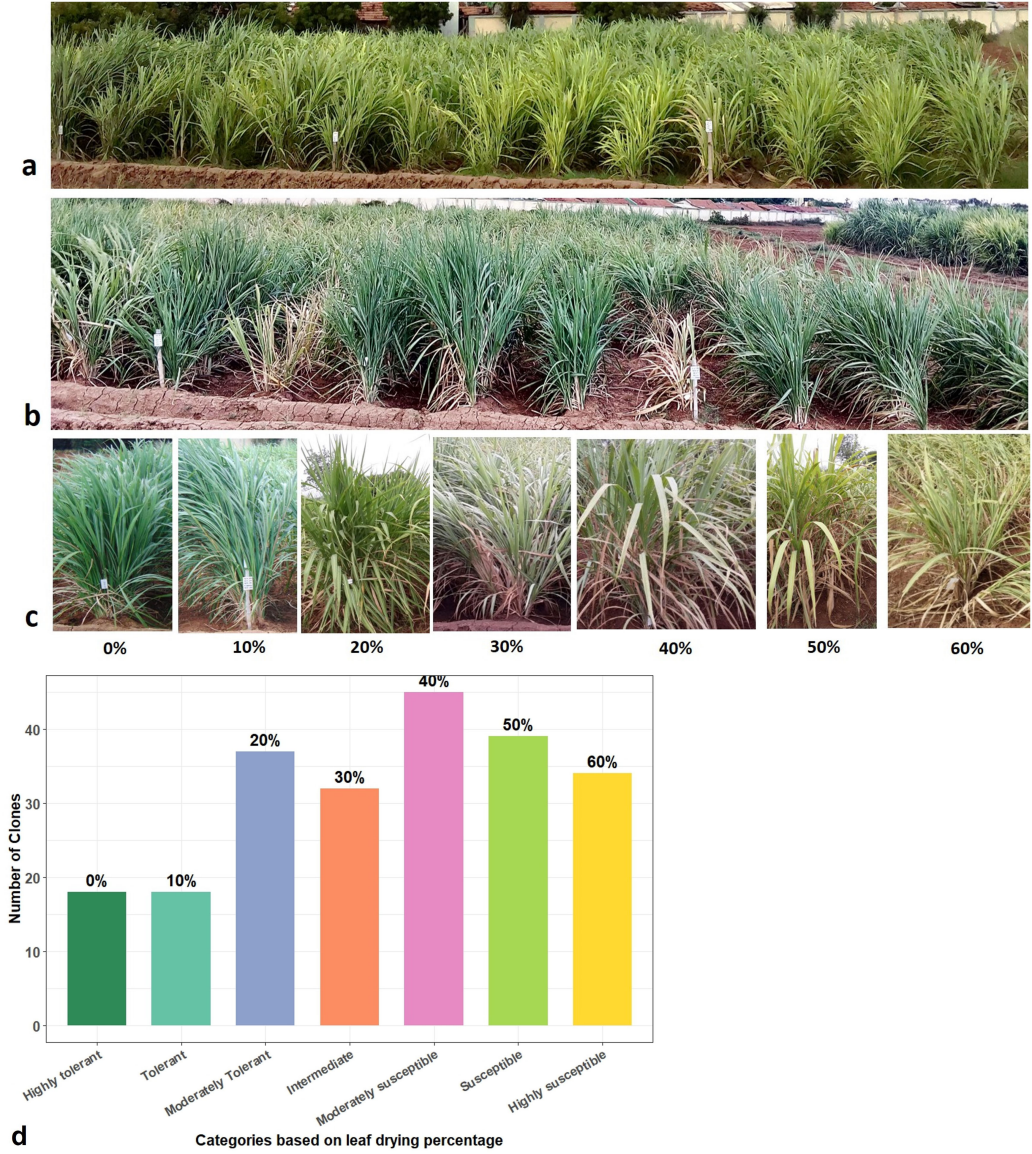
Field evaluation of *Erianthus* clones for drought tolerance response. **(a)***Erianthus* clones under irrigated control conditions. **(b)** Erianthus clones showing leaf drying symptom under drought stress conditions. **(c)** Leaf drying percentage and morphology. **(d)** Distribution of germplasm accessions grouped based on leaf drying.

**Figure 2:**
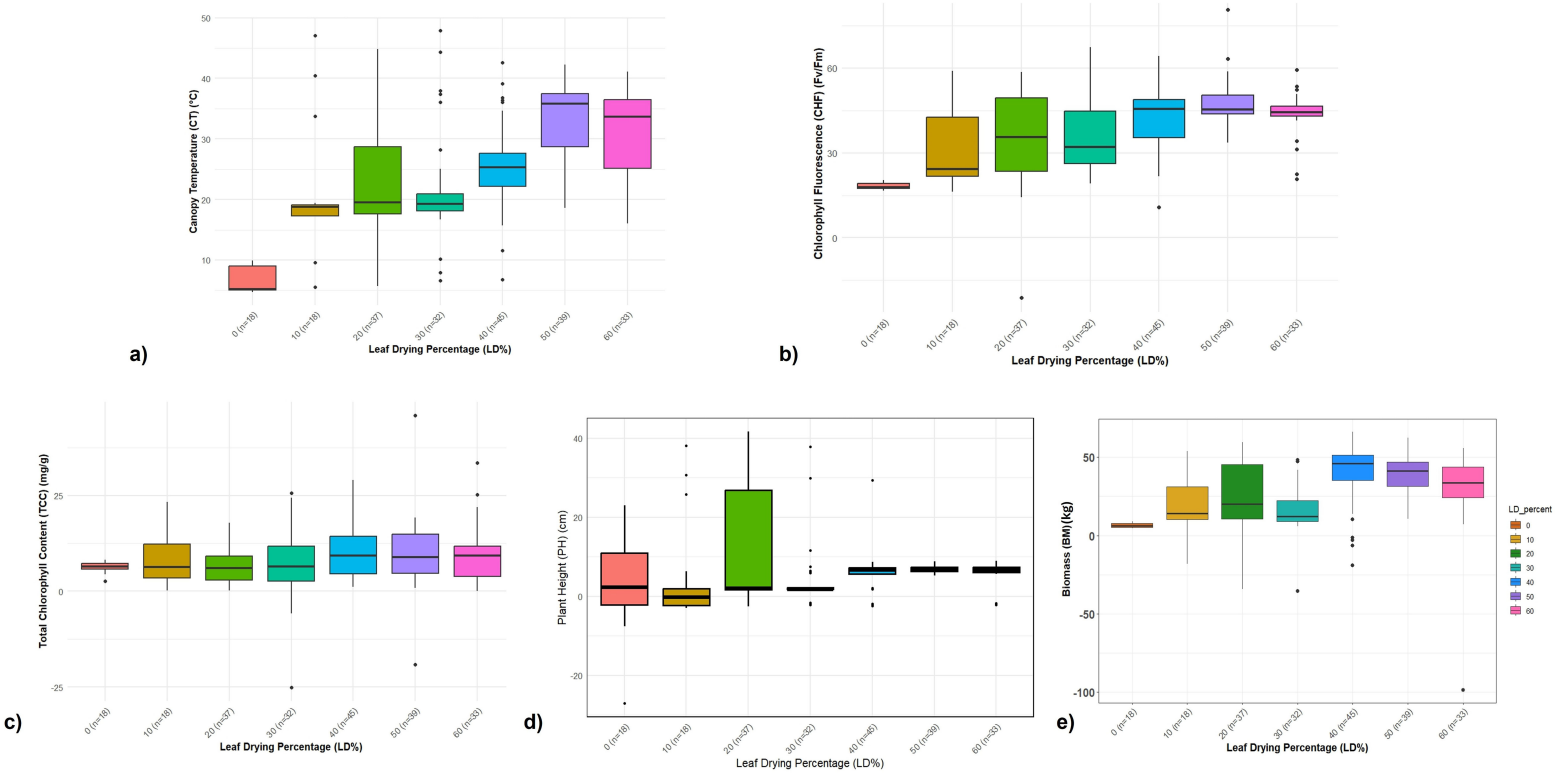
Categorization of germplasm accession based on drought tolerance and trait analysis of germplasm based on leaf drying percentage for the 2-years ABD trial. **(a–e)** Boxplot showing the relationship between different physiological traits among *Erianthus* clones categorized based on leaf drying percentage under drought stress. **(a)** Percent increase in canopy temperature. **(b)** Percent reduction in chlorophyll fluorescence (*F*_v_/*F*_m_). **(c)** Percent reduction in total chlorophyll content. **(d)** Percent reduction in plant height. **(e)** Percent reduction in biomass among *Erianthus* clones under drought stress relative to irrigated control conditions.

During moisture stress at below 60% field capacity in the ABD trial, leaf drying followed by leaf rolling were the first symptoms observed in a few *Erianthus* clones. At <50% field capacity, the clones were rated from tolerant to susceptible based on the clearly visible leaf drying symptom on the scale of 0%–60% (**Figures 1a, b**). At soil moisture below 40% field capacity, 18 clones were scored as highly tolerant (no leaf drying) or tolerant (10% leaf drying), 20% for moderately tolerant clones, 37 clones with 20% as moderately tolerant, 32 with 30%–40% as moderately susceptible, 39 clones with more than 50% leaf drying as susceptible, and 34 clones were highly susceptible with 60% leaf drying (**Figures 1c, d**). The mean percentage increase in canopy temperature and the percentage reduction in chlorophyll fluorescence (*F*_v_/*F*_m_) and total chlorophyll content under drought stress compared to control conditions corresponded to the leaf drying scores (**Figures 2a–c**; **Supplementary Figure 1**).

The increase in canopy temperature was 2%–15% lower among the highly tolerant and tolerant clones, while the clones in the intermediate and susceptible category showed a 20%–25% and 28%–47% increase, respectively. Canopy temperature was in the range of 23–24°C in control as well as highly tolerant clones under drought conditions while the susceptible clones recorded higher (29–35°C) canopy temperature. The mean reduction in chlorophyll fluorescence among the tolerant clones ranged from 8% to 25%, while the susceptible clones showed a 30%–60% reduction. A similar trend was observed for total chlorophyll content where the tolerant clones maintained a high chlorophyll content with minimum reduction at 6.25%–12%. The physiological performance of the tolerant clones was reflected in the final biomass data, with minimum reduction in plant height and biomass (**Figures 2d, e**). Highly tolerant and tolerant clones did not show any reduction in plant height, while the moderately tolerant and susceptible clones showed 15%–22% reduction compared to that of control. Mean biomass per clump ranged from 83.25 to 9.55 kg with a percentage reduction ranging from 5% to 16% among the tolerant clones and 30% to 52% among the susceptible clones.

**Figure 3:**
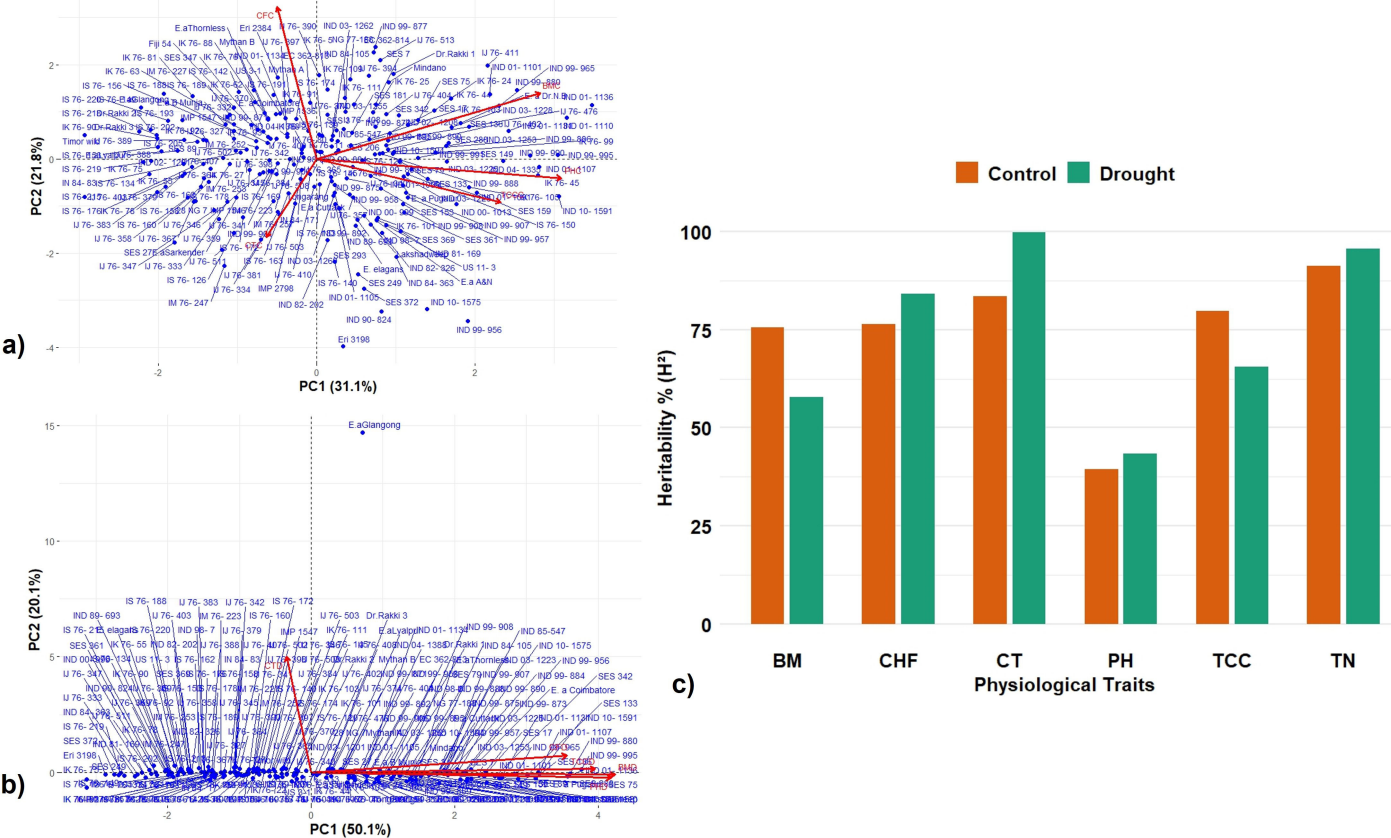
Principal component analysis (PCA) of germplasm accessions and trait relationship and heritability index of physiological traits in *Erianthus* clones under control and drought conditions. Biplot of the first two principal components of 223 germplasm accessions among the studied traits under control **(a)** and drought **(b)**. PC1 (Dimension 1), PC2 (Dimension 2). Broad sense heritability % (*H*^2^) of different physiological traits under control and drought conditions **(c)**.

PCA was performed based on mean data obtained from BLUP across 2 years’ data on canopy temperature (CTC), total chlorophyll content (TCC), chlorophyll fluorescence (CFC), plant height (PHC), and biomass (BMC) in 223 *Erianthus* accessions. The distribution and associations between various traits are depicted in a biplot against the first two principal components PC1 and PC2 (**Figures 3a, b**). The first two principal components collectively explained 52.9% of the overall variability under control conditions compared to 70.18% under drought conditions, highlighting a more concentrated distribution of variation under drought conditions. The distribution of accessions was relatively well-spread under control conditions (**Figure 3a**), while under drought conditions, it was more compressed (**Figure 3b**), and trait expression was influenced mostly by the first dimension. The traits were grouped into three major vectors under control conditions and into two groups under drought conditions. Under both conditions, chlorophyll fluorescence, biomass, and plant height showed positive association and more contribution to the first principal component. Under control conditions, canopy temperature and chlorophyll fluorescence primarily contributed more to the second principal component, whereas under drought conditions, only canopy temperature did so. The PCA biplot of 223 *Erianthus* germplasms clearly clustered tolerant and susceptible genotypes. The broad-sense heritability (*H*^2^) estimates were generally higher and more for all the traits except chlorophyll fluorescence under drought conditions (0.60–0.99) compared to the control (0.39–0.96) (**Figure 3c**).

### Phenotypic performance of the drought association panel

**Figure 4:**
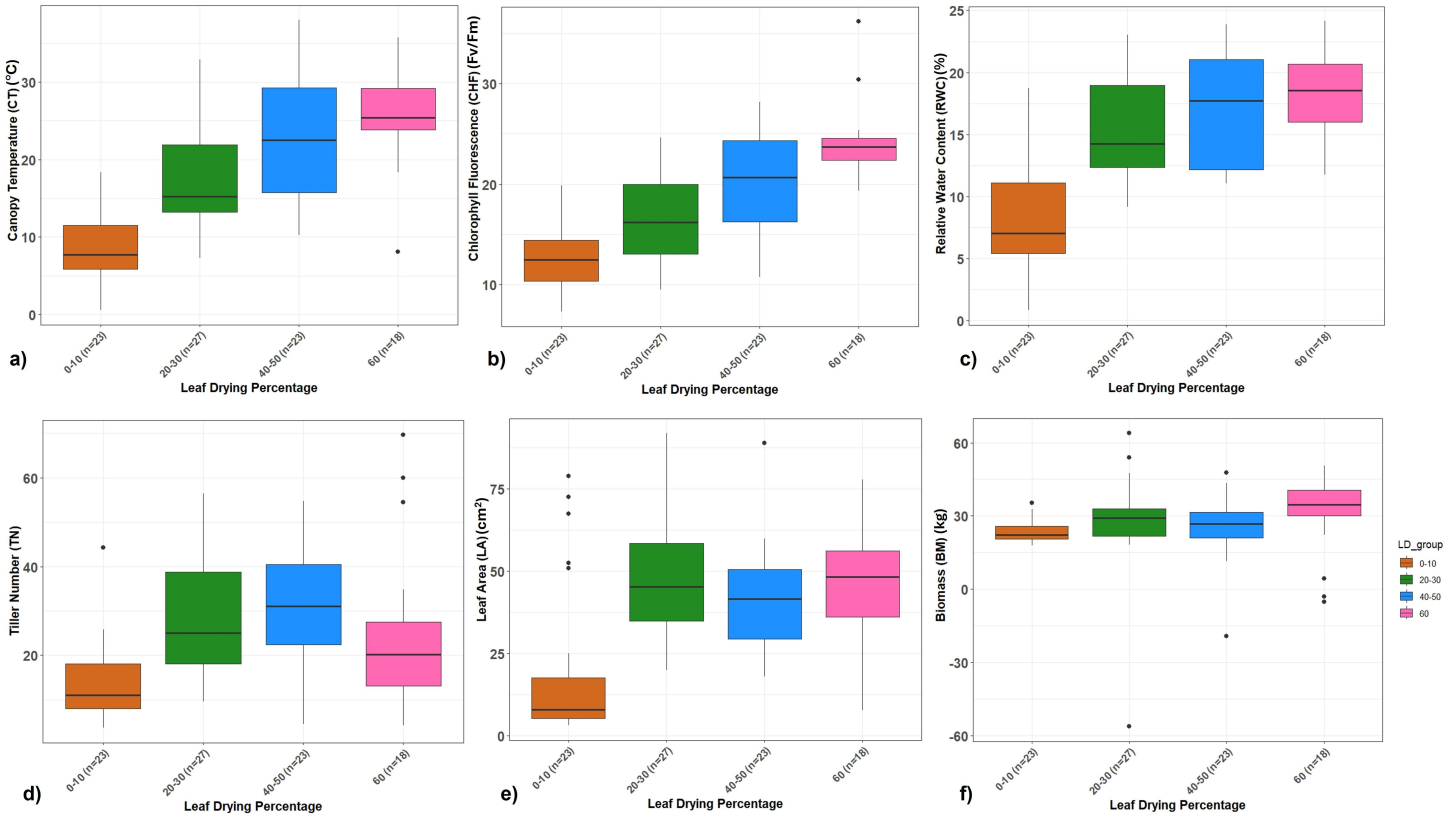
**(a–f)** Boxplot showing the relationship between different traits vs. leaf drying percentage for the 3-year RBD trial. **(a)** Percent increase in canopy temperature. **(b)** Percent reduction in chlorophyll fluorescence (*F*_v_/*F*_m_). **(c)** Percent reduction in relative water content. **(d)** Percent reduction in tiller number. **(e)** Percent reduction in leaf area. **(f)** Percent reduction in biomass of *Erianthus* clones under drought stress relative to irrigated control conditions.

The association panel consisting of 91 accessions with varying drought responses was drought-stressed during the formative phase where the respective controls were irrigated regularly. Approximately 23 clones were highly tolerant with 0%–10% leaf drying, 27 clones were tolerant with 20%–30%, 23 clones with 40%–50%, and 18 clones were highly susceptible, showing more than 60% leaf drying. The clones showed significant differences for canopy temperature, chlorophyll fluorescence (*F*_v_/*F*_m_), RWC, and total chlorophyll content under drought stress where the mean difference showed reduction compared to control conditions (**Figure 4**; **Supplementary Figure 2**). However, the reduction was significantly lower among the tolerant clones and higher in the susceptible clones. Highly tolerant clones maintained low canopy leaf temperature in the range of 23–26°C with a mean increase from 6% to 12%, while most susceptible clones recorded a leaf temperature of 30–32°C with a mean increase of 24%–28% under drought stress (**Figure 4a**; **Supplementary Figure 2**). The tolerant clones maintained high chlorophyll fluorescence (*F*_v_/*F*_m_) with 9%–18% mean reduction, while the susceptible clones showed more than 20% reduction (**Figure 4b**). Similarly, highly tolerant and tolerant clones showed lower mean reduction in leaf RWC (6%–18%), tiller number (7%–18%), and LA (5%–19%) under drought conditions (**Figures 4c–e**). The physiological performance of clones corresponded with the mean reduction in biomass yield at harvest. The tolerant clones showed 18%–22% less biomass whereas the susceptible clones had more than 42% less final biomass under drought conditions (**Figure 4**). Among the highly tolerant clones, five clones (SES 133, SES 149, SES 288, SES 293 and SES 347) consistently exhibited drought tolerance by maintaining higher chlorophyll fluorescence, RWC, leaf area, lower canopy temperature and increased biomass accumulation combining both the trials for five-years.

**Figure 5:**
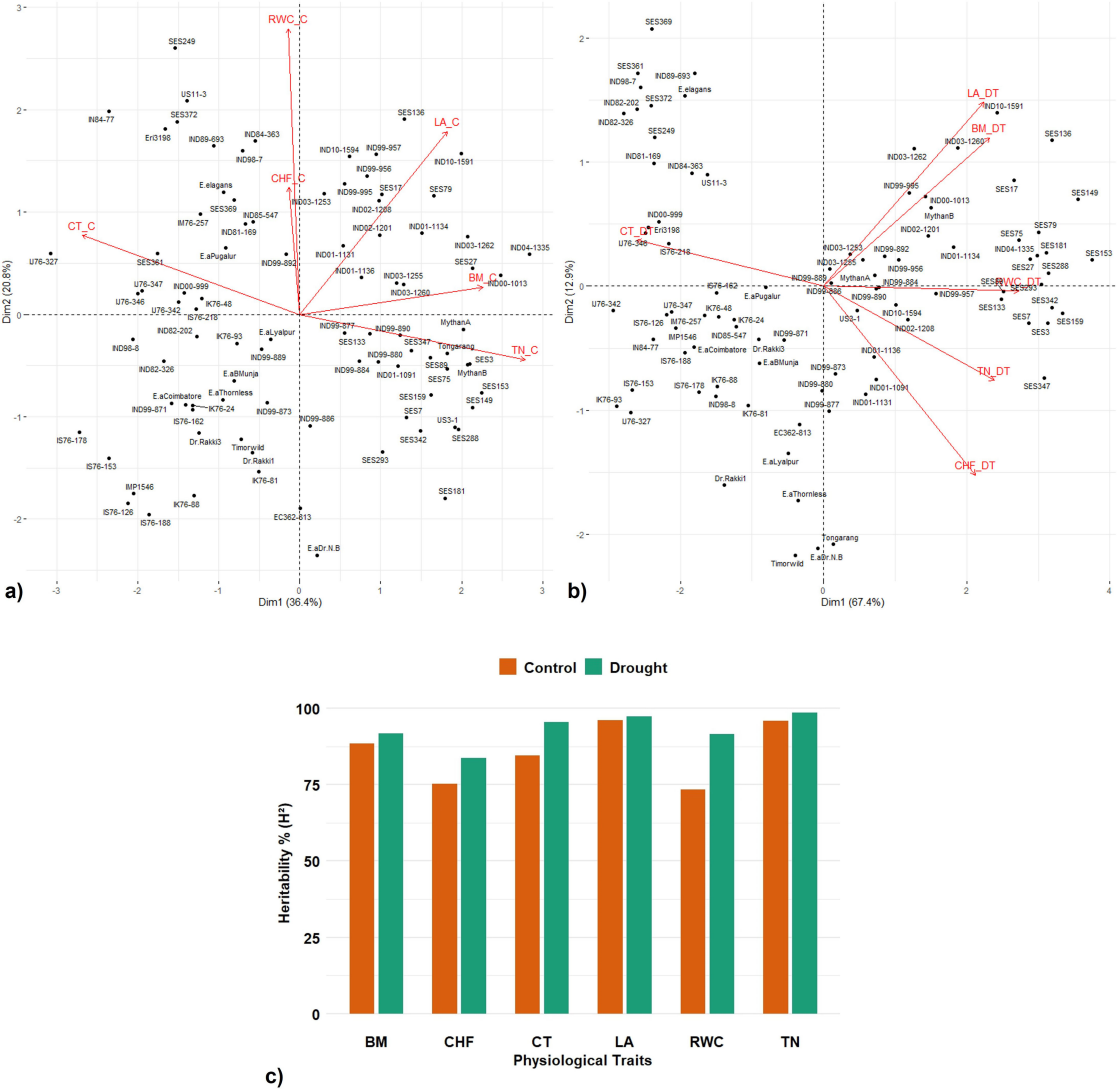
Principal component analysis (PCA) of germplasm accessions and trait relationship and heritability index under control and drought conditions. Biplot of the first two principal components of 91 accessions among the traits under control **(a)** and drought **(b)**. PC1 (Dimension 1), PC2 (Dimension 2). **(c)** Comparative broad sense heritability estimates of various traits under control and drought conditions. TN—tiller number, CF—chlorophyll fluorescence, CT—canopy temperature, LA—leaf area, RWC—relative water content, and BM—Biomass.

The PCA revealed a distinct pattern of variation in the association panel under control and drought conditions (**Figures 5a, b**). The first two principal components explained 57.2% of the total variation under control conditions with an eigen value of more than one. The accessions were widely spread, indicating wide variation in trait expression. LA, biomass, and tiller number were positively associated traits with a significant contribution to total variation. Under drought conditions, the first principal component alone explained 67.4% out of the 80.3% variation captured by the first two principal components with an eigen value of more than one. Majority of the traits including canopy temperature, chlorophyll fluorescence, LA, RWC, and biomass showed positive correlation with a greater contribution to the first principal component. Canopy temperature was the sole significant contributor to the second principal component under drought conditions. PCA clearly clustered the tolerant and susceptible clones under both control and drought stress conditions. The heritability percent estimated for various physiological traits were in the range of 74%–96% under control conditions and in the range of 86%–99% under drought conditions; tiller number showed the maximum heritability percentage under both control and drought conditions (39-96) (**Figure 5c**).

### Stress tolerance and susceptibility indices of clones in the association panel

**Figure 6:**
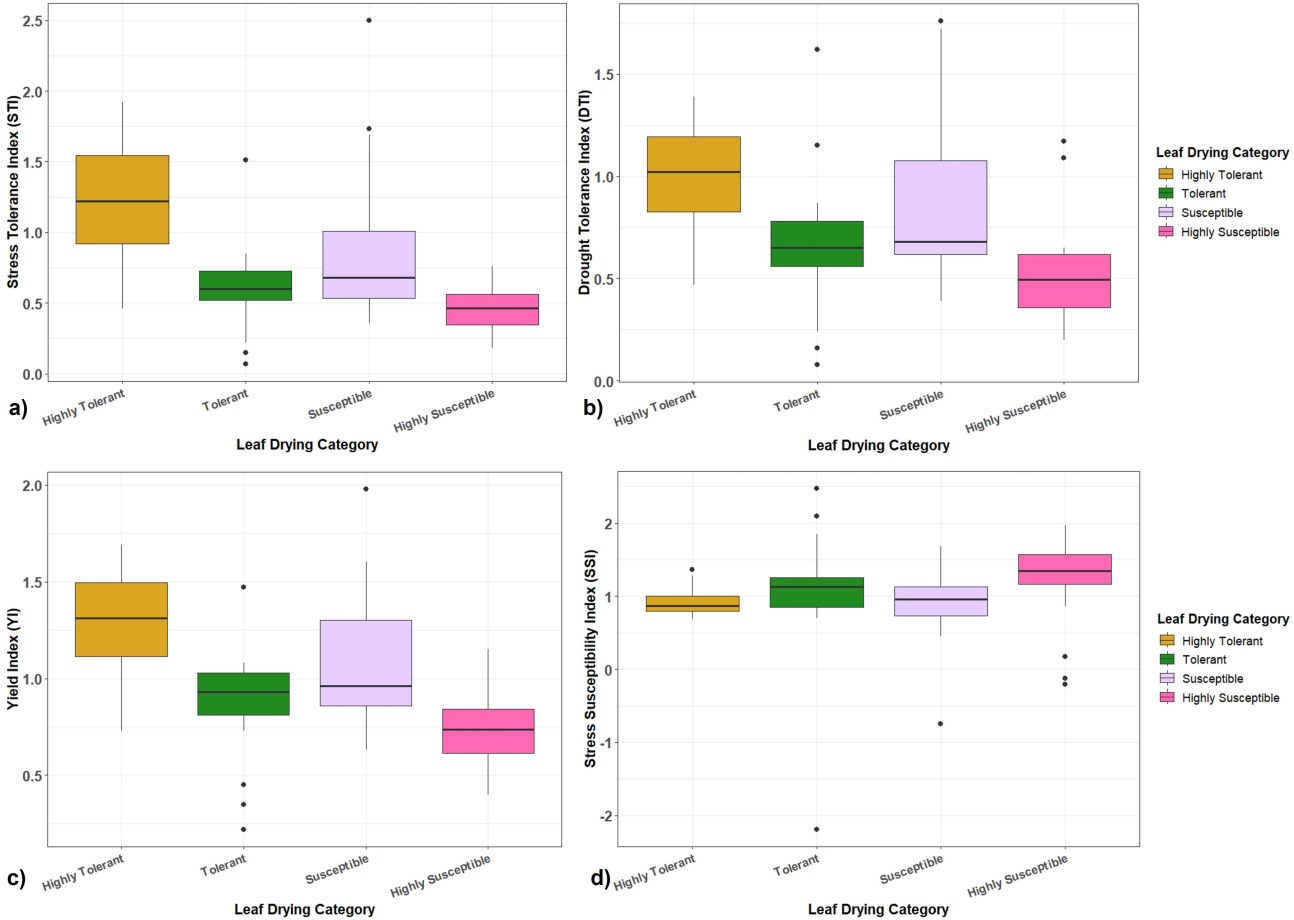
Boxplots showing the relationship between different drought tolerance indices with highly tolerant, tolerant, susceptible, and highly susceptible categories of *Erianthus* clones. **(a)** Stress tolerance index (STI). **(b)** Drought tolerance index (DTI). **(c)** Yield index (YI). **(d)** Stress susceptible index (SSI).

The performance of *Erianthus* clones in the association panel with respect to final biomass under drought stress showed that the STI ranged from 0.07 to 2.50 with a grand mean of 0.79 (**Figure 6a**) with 24 (highly) tolerant clones having higher STI (>1.01) for biomass compared to the susceptible clones. The DTI was in the range of 0.08 and 1.76 (**Figure 6b**) where the tolerant clones showed higher DTI (0.82 to 1.21). A total of 23 clones had a higher DTI (>1.02) while 3 clones (IND 99-892, IND 10-1591, and IND-03 1260) recorded a higher DTI than the tolerant check. The average YI calculated based on the biomass of the clones was 1.0 with a range from 0.22 to 1.98 (**Figure 6c**). Ten germplasms displayed higher YI at values more than 1.6. SSI ranged from 0.17 to 2.48 with an average of 1.02. SSI for all highly tolerant clones was less than 1.0 (**Figure 6d**). Two *Erianthus* clones (SES 288 and SES 293) with superior physiological performance recorded higher STI (1.92 and 1.87), DTI (1.39 and 1.36), YI (1.69 and 1.66) and lower SSI (0.69 and 0.70).

### Genotyping by sequencing and variant calling

GBS of 91 *Erianthus* germplasm clones generated 5.143 Gb raw data and 5.142 Gb clean data after filtering out low-quality data. The average raw data generated for the clones were 1.83 million reads, which, after QC, resulted in 1.76 million reads with a Phred score of more than 20. Mapping of cleaned short reads against the *E. rufipilus* (https://sugarcane-genome.cirad.fr/node/1/7) reference genome identified 3,696 bi-allelic SNPs common between three software. Further filtering with a minor allele frequency set at 5% and 20% missing genotype resulted in 1,044 SNPs that were used for downstream marker–trait association.

### Population structure and linkage disequilibrium

**Figure 7:**
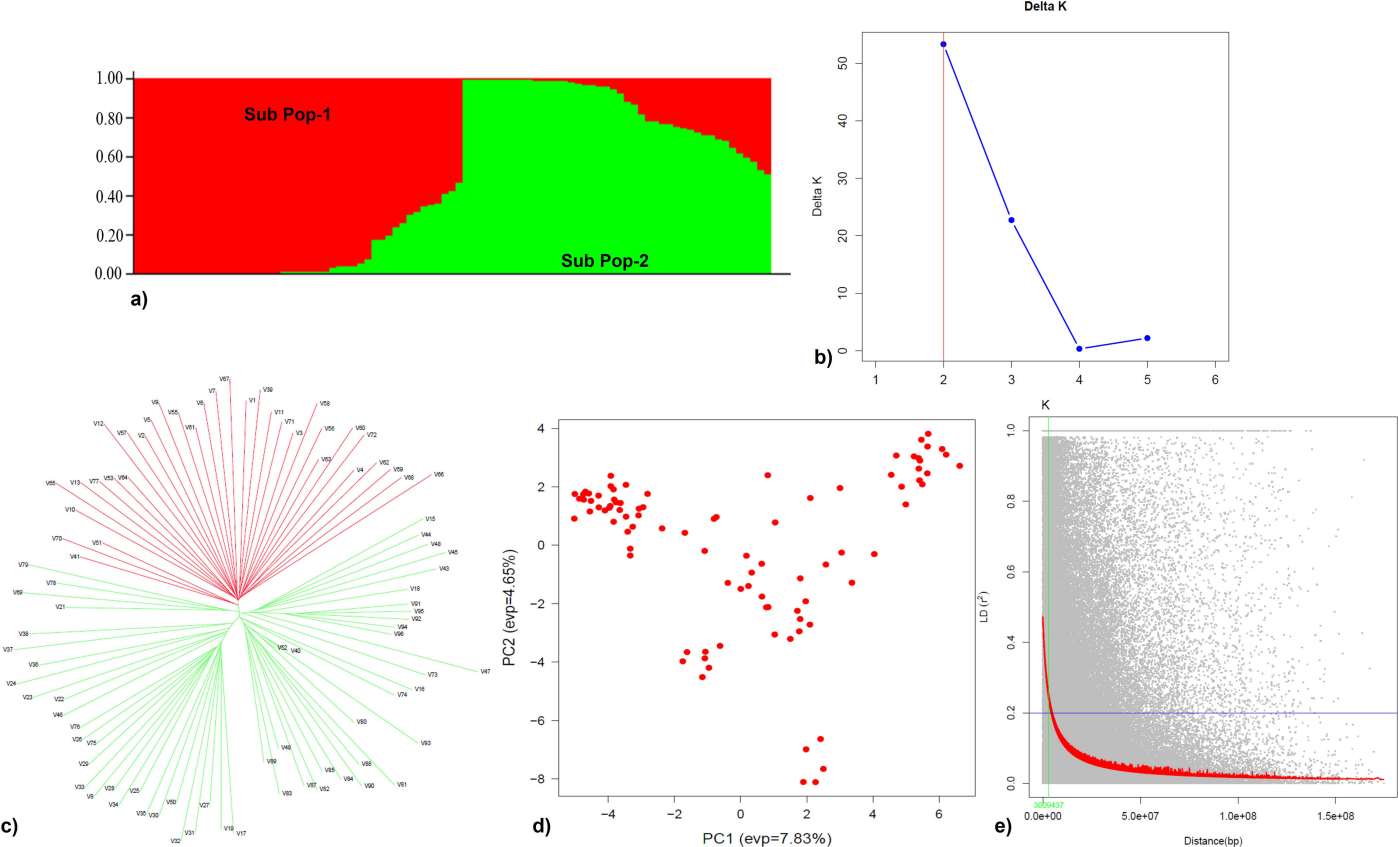
Population structure, principal component, and linkage disequilibrium analyses of the *Erianthus* drought panel. **(a)** Bar plot representing two subpopulations (Sub_pop 1 and Sub_pop 2). **(b)** Delta K plot—Estimation of optimum number for subpopulation (highest peak at *K* = 2). **(c)** Neighbor-joining tree of 91 germplasm accessions illustrating the separation of studied germplasm accessions into two main clusters corresponding to structure analysis. **(d)** Scatter plot of the first two principal components (PC1 and PC2) showing variation present in the accessions. **(e)** Scatter plot showing the LD decay with physical distance (bp); red color indicates the fitted LD decay.

Population structure analysis, based on the Δ*K* method, categorized 91 genotypes into two sub-populations, SP1 and SP2, consisting of 43 and 48 genotypes, respectively. Individuals of each population were further categorized into two groups, i.e., pure and admixture types, where populations comprising ≥0.8 of the member proportions were considered pure and others as admixtures. Based on this criterion, SP1 was 75% pure and 25% admixtures and SP2 was 63% pure and 37% admixtures (**Figures 7a, b**). Similar results were found by Tsuruta et al. (2022) where the high values of Δ*K* were obtained at *K* = 2 and *K* = 3 with 121 *Erianthus* accessions from Thailand based on the marker profiles with 28 SSR primers. The tree based on the neighbor-joining method also identified two clusters (**Figure 7c**), indicating consistency in the grouping of genotypes. From the PCA, PC1 explained 32.8% of the genetic variance while PC2 explained 13.0% (**Figure 7d**), which distinguished the two sub-populations based on their geographic regions. The background LD in the analyzed association mapping panel (AM) was equal to 0.2, which was taken as the threshold cutoff for estimating LD decay. The whole-genome LD in the AM panel decayed at 3 Mb (**Figure 7e**).

### Genome-wide association analysis

**Figure 8:**
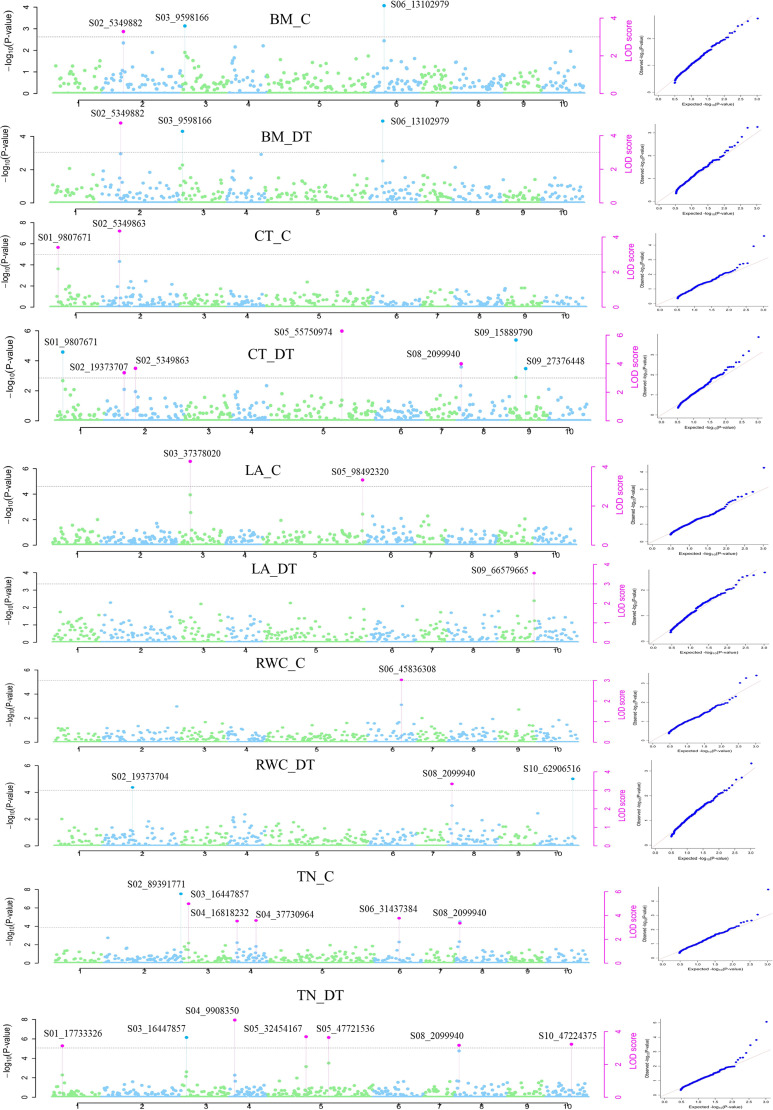
Manhattan plots showing significant QTN (purple color coded) with the corresponding QQ (quantile–quantile) plots representing the distribution of expected and observed *p*-values for various traits under control and drought conditions. CT_C and CT_ DT—Canopy temperature under control and drought conditions, respectively. LA_C and LA_DT—Leaf area under control and drought conditions, respectively. RWC_C and RWC_DT—Relative water content under control and drought conditions, respectively. TN_C and TN_DT—Tiller number under control and drought conditions, respectively. BM_C and BM_DT—Biomass under control and drought conditions, respectively.

Genome-wide association mapping using six MrMLM models identified 43 QTNs distributed over 10 chromosomes associated with 12 traits under control and drought conditions. Among the six models, ISIS EM-BLASSO and pLARmEB revealed the maximum number of QTNs, i.e., 30 and 22, respectively, whereas FASTmrEMMA detected the lowest number (10) of QTNs. Similarly, 46 QTNs in 10 chromosomal regions were detected using four models in GAPIT. Of these QTNs, 23 were common in at least two models (**Table 1**; **Figure 8**). The phenotypic variation explained (PVE or *R*^2^) by the QTNs ranged from 0.26% to 59.35%, indicating that traits under both control and drought conditions were controlled by multiple loci with small to moderate effects. The most significant QTN was recorded for biomass under drought conditions (BM_DT) linked to marker *S02_5349882* at –Log_10_*p*-value = 6.2 and *R*^2^ = 54.1% followed by tiller number under drought (TN_DT) linked to *S04_9908350* at –Log10 *p*-value = 6.9 and *R*^2^ = 20.1%. The highest number of QTNs was identified on Chr2 (22) followed by Chr6 (17) (**Table 1**; **Figure 8**). Three major QTNs (*S02_5349882* identified by five models, *S03_16447857* by four models, and *S04_9908350* by five models) were identified in Chr2, Chr3, and Chr4, respectively. Trait-wise, QTNs ranged from one each for RWC under control (RWC_C) and LA under drought (LA_DT) conditions to seven QTNs associated with tiller number (TN_DT) as well as canopy temperature (CT_DT) under drought conditions (**Table 1**). Altogether, 26 QTNs were identified to be associated with different traits under drought conditions whereas 17 QTNs were identified for traits under control conditions. Seven QTNs were common between control and drought conditions, while 9 and 17 QTNs expressed specifically under control and drought conditions, respectively. One QTN (*S03_16447857*) was associated with both LA (LA_C) and tiller number (TN_C) under control conditions. Moreover, two QTNs (*S05_47721536* and *S08_2099940*, both with FDR *p*-value = 0.013) identified by MrMLM as well as the GAPIT model were associated with TN_DT. On the other hand, one QTN (*S08_2099940*) showed association with more than one trait (CT_DT, RWC_DT, and TN_DT) under drought stress (**Table 1**).

**Table 1:** Quantitative trait nucleotides (QTNs) associated with various traits under control and drought conditions in the *Erianthus* drought panel.

Traits	QTNs	Chr	Position (bp)	Model	P value	PVE/R^2^ (%)	LOD score	Effect	MAF
BM_C	S02_5349882	2	5349882	1,2,4,6, 9	3.9-4	19.9-45.7	3.27-3.29	3.2	0.13
BM_C	S03_9598166	3	9598166	5	4.3	19.5	3.58	-8.7	0.13
BM_C	S06_13102979	6	13102979	5	5.4	31.8	4.66	10.1	0.31
BM_DT	S02_5349882	2	5349882	1,2,3,4,5	5.3-6.2	35.3-54.13	4.6-5.4	14.1	0.13
BM_DT	S03_9598166	3	9598166	4	5.01	23.12	4.25	-9.2	0.13
BM_DT	S06_13102979	6	13102979	4	5.65	29.21	4.86	9.4	0.31
CT_C	S02_5349863	2	5349863	1,2,3,4,5,6	3.9-6.7	21.4-59.3	3.2-5.9	2.8	0.23
CT_C	S01_9807671	1	9807671	3,5	3.7-4.5	17.7	3-3.7	0.9	0.12
CT_DT	S02_5349863	2	5349863	1,2	3.8-4.3	21.1-24.6	3.1-3.5	2.4	0.23
CT_DT	S02_19373707	2	19373707	1,2	3.8-4.9	16.4-18.5	3.1-4.2	2.1	0.25
CT_DT	S08_2099940	8	2099940	1,2,3,4,5,7,8,9,10	4.1-6.7	12.8-45.5	3.4-5.9	-1.8	0.14
CT_DT	S05_55750974	5	55750974	4,5	6.7-7.5	14.7-21.9	5.9-6.6	-1.7	0.39
CT_DT	S01_9807671	1	9807671	5	5.6	11.1	4.8	1.7	0.12
CT_DT	S09_15889790	9	15889790	5	6.482	20.8	5.7	-2.1	0.20
CT_DT	S09_27376448	9	27376448	5	4.3919	14.8	3.7	1.8	0.27
CHF_C	S01_102019624	1	102019624	1,3	3.8-4.5	35.9	3.1-3.7	0.02	0.24
CHF_C	S05_79251361	5	79251361	5	3.8-4.5	17.8-25	3.1-3.7	1.00E-02	0.18
CHF_C	S02_50417983	2	50417983	1,2	4.8409	10.02	4.0852	0.008	0.24
CHF_DT	S06_37343127	6	37343127	6	5.0071	49.46	4.2438	-0.042	0.31
CHF_DT	S03_493324	3	493324	5	6.3419	7.73	5.5256	0.018	0.25
CHF_DT	S05_55750756	5	55750756	5	6.314	28.01	5.4987	0.0351	0.31
CHF_DT	S05_74779768	5	74779768	5	6.3216	1.22	5.506	0.0084	0.12
CHF_DT	S10_29330993	10	29330993	5	6.0604	0.26	5.2543	0.0035	0.21
LA_C	S03_37378020	3	37378020	1,2,4,5,6	4.5-5	23.8-43.4	3.7-4.2	-60.1582	0.13
LA_C	S05_98492320	5	98492320	2,4,9	4.04	0.5-0.7	3.32	39.5	0.23
LA_DT	S09_66579665	9	66579665	1,2,4,5,6	4.2	20.1-46.6	3.5	70.34	0.34
RWC_C	S06_45836308	6	45836308	1,2,3,4,5,7,8,9,10	3.7-5.1	7-27.4	3.03-4.4	-1.0253	0.46
RWC_DT	S08_2099940	8	2099940	1,3,4,6	3.9-5	16.9-42.2	3.2-4.2	4.2	0.14
RWC_DT	S02_19373704	2	19373704	4	3.8	10.4	3.1	2.4	0.19
RWC_DT	S10_62906516	10	62906516	5	4.3	9.6	3.6	1.7	0.48
TN_C	S03_16447857	3	16447857	1,2,4,5	5.7-5.9	9.4-16.5	4.9-5.1	5.1	0.42
TN_C	S04_16818232	4	16818232	1,2,4,5,6	4.2-5.8	15.4-25.4	3.1-5	6.3	0.25
TN_C	S08_2099940	8	2099940	1,2,3,4,5,6	3.7-4.9	13.5-36	3-4.2	7.1	0.14
TN_C	S04_37730964	4	37730964	4,5	3.8-4.7	5.8-10.1	3.1-4	4.7	0.42
TN_C	S06_31437384	6	31437384	4,5, 9,10	4.1-4.8	9.8-9.9	3.4-4.1	-3.9	0.20
TN_C	S02_89391771	2	89391771	6	6.6429	20.54	5.8162	-6.7	0.36
TN_DT	S04_9908350	4	9908350	1,2,4,5,6	5.1-6.9	16.4-20.1	4.3-6.1	6.3	0.34
TN_DT	S05_32454167	5	32454167	1,2,3,4,5,6	3.8-4.8	18.3-42.9	3.1-4.1	-5.6	0.18
TN_DT	S03_16447857	3	16447857	3	4.4	17.9	3.6	10.4	0.41
TN_DT	S01_17733326	1	17733326	4,5	3.8	6.5	3.1		0.26
TN_DT	S05_47721536	5	47721536	4,5,7,8,9,10	4.3	10.5	3.6	-4.5	0.15
TN_DT	S08_2099940	8	2099940	4,5	3.8	11.7-11.9	3.1	5.0	0.14
TN_DT	S10_47224375	10	47224375	4,5	3.9	4.9	3.2	-4.8	0.45

1-mrMLM, 2- FASTmrMLM, 3-FASTmrEMMA, 4-pLARmEB, 5-ISIS EM-BLASSO,6-pKWmEB,7-MLMM,8-FarmCPU,9-BLINK, 10-CMLM; bp, base pair; BM, biomass; Chr, chromosome; CT, canopy temperature; CHF, chlorophyll fluorescence; LA, leaf area; MAF, minor allele frequency; PVE/R^2^, phenotypic variance explained; RWC, relative water content; TN, tiller number.

### Candidate genes identified in the QTNs

A total of 235 genes were identified linked to 34 QTNs associated with different traits under drought stress, of which 34 genes were unique and 27 have previously known functional annotations (**Supplementary Tables 1**, **2**). Maximum candidate genes were identified from significant QTNs associated with different traits under drought stress. QTN (*S08_2099940*) associated with three physiological traits under drought conditions (CT_DT, TN_DT, and RWC_DT) was annotated as a regulatory-associated protein of TOR 2 (Target of Rapamycin 2). Several candidate genes such as potassium transporter (QTN-*S06_37343127*), auxin response factor (QTN-*S05_55750756*), FAD-binding PCMH-type domain-containing protein (QTN-*S03_493324*), protein kinase domain-containing protein (QTN-*S05_74779768*), and EG45-like domain-containing protein (QTN-*S10_29330993*) were identified from QTNs associated with chlorophyll fluorescence under drought conditions (CF_DT). Two candidate genes encoding receptor protein kinase TMK1 (Trans-Membrane Kinase 1) (QTN-*S01_17733326*) and putative NRT1/PTR (Nitrate Transporter 1/Peptide Transporter Family) family protein (QTN-*S10_47224375*) were identified to be associated with TN_DT. Two candidate genes such as putative pentatricopeptide repeat-containing protein and putative polyubiquitin were identified from QTNs linked to markers *S02_19373704* and *S10_62906516*, respectively, for RWC_DT.

## Discussion

Sugarcane achieves 70%–80% of yield during its formative phase while the remaining yield is attained during the grand growth phase. Drought stress during these critical growth stages with high water requirement (Hemaprabha et al., 2013; Reyes et al., 2021) severely affects cane growth, yield, and sucrose content (Silva et al., 2007; Ferreira et al., 2017) with up to 50%–60% yield reduction depending on the severity and number of drought spells (Vinoth et al., 2022). Most of the commercial sugarcane genotypes with a narrow genetic base show poor tolerance to drought (Hemaprabha et al., 2013; Mohanraj et al., 2021). Therefore, there is a need for alternative sources of resistance to facilitate the development of sugarcane genotypes that can not only withstand the extreme environmental stress but also sustain yield. *Erianthus* has been identified as a sugarcane-related genus with many significant drought-adaptive morphological and physiological traits (Valarmathi et al., 2023). Extensive phenotyping and identification of the sequence variations in *Erianthus* spp. controlling critical agronomic traits under drought conditions could help identify resources to improve sugarcane productivity in drought-prone areas.

### Higher photosynthetic efficiency among tolerant *Erianthus* clones

*Erianthus* clones employ several important physiological and biochemical traits to adapt and mitigate the effect of water stress (Valarmathi et al., 2023). Maintaining higher photosynthetic efficiency, cooler canopy, and higher RWC; accumulating osmotic solutes; and enhancing water uptake through its deep root system, *Erianthus* clones thrive under water stress with high water use efficiency to sustain plant growth and biomass production (Baby et al., 2005). Superior physiological and growth responses of clones at the formative stage of drought stress can be early indicators to select drought-tolerant genotypes with a better yield.

Drought stress impedes plant growth primarily through the reduction in photosynthetic efficiency, which serves as a reliable prime physiological biomarker to assess drought stress response (Wang et al., 2025). Intact green canopy maintained in the highly tolerant and tolerant *Erianthus* clones shows less photosynthetic organ damage, leading to the maintenance of high photosynthetic efficiency during drought stress. Canopy leaf temperature and chlorophyll fluorescence (*F*_v_/*F*_m_) are other important physiological traits to sustain photosynthetic activity under drought stress. Normal transpiration maintains the canopy temperature at a metabolically functional range, while closure of stomata during high evaporative demands under drought stress leads to a high canopy temperature (Siddique et al., 2000). The lower canopy temperature recorded among tolerant genotypes could be due to the efficient extraction of available soil moisture by their deep root system to maintain normal transpiration and transpirational cooling (Reynolds et al., 2010). Chlorophyll fluorescence (*F*_v_/*F*_m_), an important photochemical quenching parameter that indicates the quantum efficiency of PSII and reduction in *F*_v_/*F*_m_, suggests downregulation of photosynthesis due to the inactivation of PSII activity (Yao et al., 2018). Along with chlorophyll fluorescence, reduction in RWC and chlorophyll content is also associated with decreased photosynthetic efficiency (Kumar et al., 2024). The tolerant *Erianthus* genotypes with a higher chlorophyll fluorescence, RWC, and chlorophyll content were able to regulate photosynthetic rates as well as transpirational cooling under water stress conditions, which supported growth and final biomass accumulation.

### Higher drought tolerance indices recorded for tolerant *Erianthus* clones

Stress tolerance indices provide a measure of drought effects to identify drought-tolerant genotypes where genotypes with higher drought indices are more likely to be tolerant and stable under stress conditions (Wirojsirasak et al., 2023). Different drought tolerance indices across *Erianthus* germplasms revealed significant differences in their adaptability under drought stress to sustain biomass production. Highly tolerant and tolerant clones showing consistently higher tolerance indices (STI and DI) and lower SSI indicate the ability of these clones to sustain growth and biomass production with minimal reduction under drought stress (Augustine et al., 2015; Sanghera and Kashyap, 2022). The susceptible genotypes with a high SSI and a poor STI score supported their significant stress-induced biomass reduction.

### Marker–trait association and linked genes

Genome-wide association analysis detected a total of 43 QTNs distributed on 10 different chromosomal regions, which explained between 0.26% and 59.35% of the total phenotypic variation, indicating that the traits were controlled by multiple small–moderate effect loci. The 17 QTNs that were uniquely identified under drought stress are important for consideration of further validation using the genotypes that were not used for this GWAS. Of special importance are the two significant QTNs (S05_47721536 and S08_2099940) for the tiller number under drought conditions, specifically the pleiotropic QTN (S08_2099940) that was significantly associated with more multiple traits such as canopy temperature, RWC, and tiller number under drought stress. Both QTNs resided in the intergenic region of the genome with a modifier effect on the traits.

Of the 34 unique genes from 235 genes identified from drought stress-responsive traits associated with 33 QTNs, 27 genes had known functional implications in drought perception and signaling. TOR 2, linked to the pleiotropic QTN (S08_2099940), belongs to the serine/threonine protein kinase family, which regulates signaling networks involved in cell growth and development and cellular metabolism (Bakshi et al., 2021). Overexpression of TOR was shown to increase the accumulation of osmolytes such as proline to maintain cellular osmotic potential under abiotic stress conditions (Caldana et al., 2013). Candidate genes such as potassium transporter, auxin response factor, FAD-binding PCMH-type domain-containing protein, protein kinases, and EG45-like domain-containing protein were identified from QTNs associated with chlorophyll fluorescence under drought stress. Optimal potassium nutrition contributes to important biological functions to maintain osmoregulation and photosynthetic carbon metabolism when plants are exposed to drought stress conditions (Cakmak and Rengel, 2024). Genes encoding for potassium transporters and auxin response factors linked to QTNs associated with chlorophyll fluorescence in *Erianthus* clones might contribute to maintaining the photosynthetic efficiency and chlorophyll content under drought conditions (Verma et al., 2022). The receptor protein kinase TMK1 family protein identified in the study has been shown to be a key regulator of abscisic acid biosynthesis via auxin signaling under drought stress (Yang et al., 2021). NRT1/PTR family protein in the QTN associated with tiller number under drought stress is reported to increase nitrate reductase activity, contributing to increased root length and biomass production (Nedelyaeva et al., 2024).

A GWAS was performed in this study with 1,044 high-quality SNPs, which could be considered less given that the *Erianthus* clones used in the study had varying ploidy levels with 2n=2x=20 to 2n=2x=60. However, we believe that with the LD as large as ~3 Mb, the number of markers used were sufficient to capture the LD blocks. On the other hand, owing to budgetary constraints, the drought association panel investigated for GWAS was a small representative of the larger world collection of *Erianthus* clones. Although selective GBS of such a small-size population has been used to capture QTLs in sugarcane (Gutierrez et al., 2018), the use of the entire collection with more genotypic diversity will help capture better marker–trait associations.

## Conclusion

Screening of *Erianthus* clones from a diverse collection of sugarcane and the related germplasm pool identified drought-tolerant clones with significant physiological adaptation traits that were able to maintain a higher photosynthetic efficiency to sustain growth and biomass accumulation under drought conditions. These clones can be used as important genetic resources in sugarcane breeding program to improve drought tolerance. The significant QTNs, especially the QTN (S08_2099940) associated with more than one trait, upon further validation using a larger-size and a more diverse population and/or interspecific cross progeny, can be used to develop diagnostic markers to facilitate marker-assisted transfer of drought tolerance-associated alleles into high-yielding but drought-sensitive sugarcane clones for the selection of drought-tolerant clones early in the variety development process. Similarly, genes such as target of rapamycin and serine/threonine protein kinase linked to the significant QTNs can be used to improve drought tolerance in sugarcane using gene overexpression and/or knock-out strategies.

## Data Availability

The sequence data analyzed in this study have been deposited to the NCBI SRA database and are available at https://www.ncbi.nlm.nih.gov/ (Bioproject PRJNA1368756).

## Supplementary material

Supplementary Figure 1 Percentage reduction in morphological and physiological traits among 223 *Erianthus* clones under drought stress compared to irrigated control condition.

Supplementary Figure 2 Percentage reduction in morphological and physiological traits among *Erianthus* drought panel under drought stress compared to irrigated control condition.
