# Supplementary figures and images for: Identification of new genetic resources for drought tolerance-related traits from the world Erianthus germplasm collection
Source: Front Plant Sci. 2025 Nov 28;16:1684712. doi: 10.3389/fpls.2025.1684712 (PMC12699327; doi:10.3389/fpls.2025.1684712)

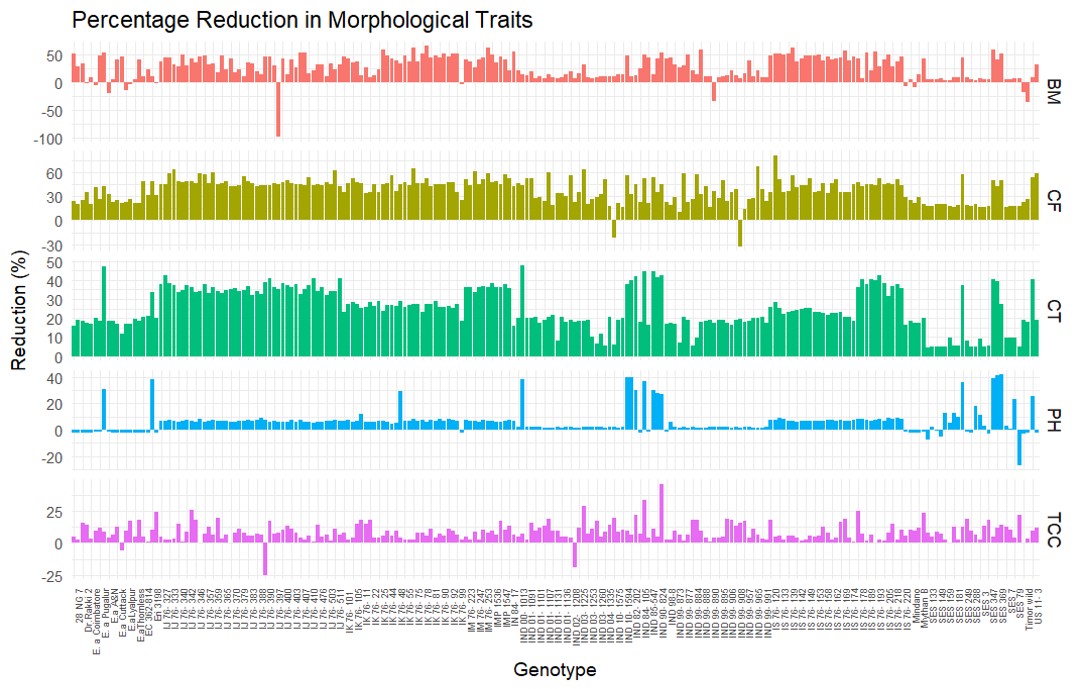

Supplement: Supplementary Figure 1 — Percentage reduction in morphological and physiological traits among 223 Erianthus clones under drought stress compared to irrigated control condition. [file Image1.jpeg]

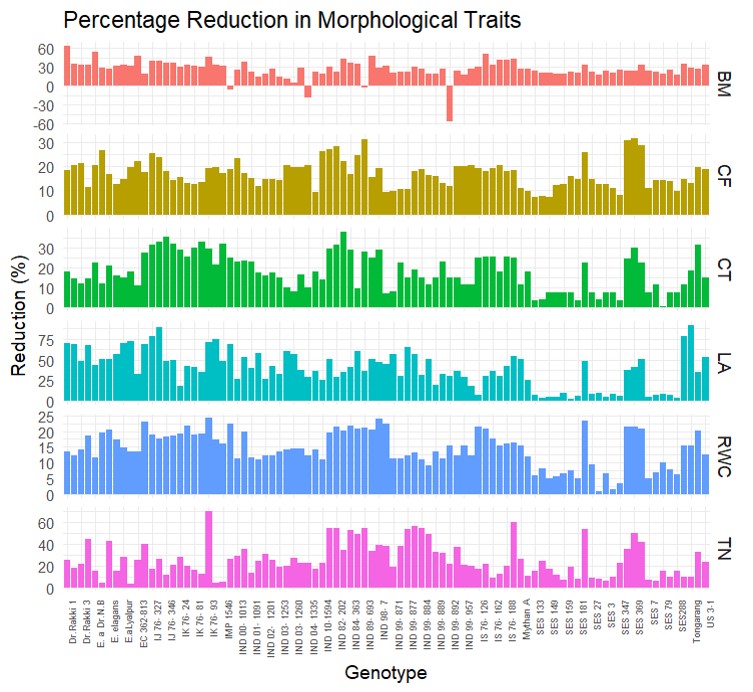

Supplement: Supplementary Figure 2 — Percentage reduction in morphological and physiological traits among Erianthus drought panel under drought stress compared to irrigated control condition. [file Image2.jpeg]
